# A Novel Immune-Related Competing Endogenous RNA Network Predicts Prognosis of Acute Myeloid Leukemia

**DOI:** 10.3389/fonc.2020.01579

**Published:** 2020-08-11

**Authors:** Shujuan Wang, Lu Yang, Yajun Liu, Yan Xu, Danfeng Zhang, Zhongxing Jiang, Chong Wang, Yanfang Liu

**Affiliations:** ^1^Department of Hematology, The First Affiliated Hospital of Zhengzhou University, Zhengzhou, China; ^2^Department of Orthopaedics, Rhode Island Hospital, Warren Alpert Medical School, Brown University, Providence, RI, United States

**Keywords:** acute myeloid leukemia, immune microenvironment, weighted gene coexpression network analysis, competing endogenous RNA network, prognosis.

## Abstract

**Background:**

Acute myeloid leukemia (AML) is a genetically, biologically and clinically heterogeneous hematopoietic malignancy that is highly dependent on the bone marrow (BM) microenvironment. Infiltrated immune cells and stromal cells are an important part of the BM microenvironment and significantly affect the progression of AML. Recently, the competing endogenous RNA hypothesis has gained great interests in the study of molecular and biological mechanisms of tumor occurrence and progression. However, research on how competing endogenous RNA relates to leukemia tumor microenvironment remains uninvestigated.

**Methods:**

In this study, mRNA, miRNA and lncRNA data and clinical information of the AML cohort were obtained from The Cancer Genome Atlas (TCGA) database, and the immune and stromal scores were calculated using the ESTIMATE algorithm.

**Results:**

We found that immune scores were significantly correlated with cytogenetic risk and overall survival, and also identified microenvironment-related mRNAs, miRNAs, and lncRNAs based on the immune and stromal scores. Differentially expressed mRNAs and lncRNAs were applied to weighted correlation network analysis (WGCNA) to identify the modules most relevant to the immune microenvironment of AML. Using miRNA database to predict miRNA-targeted genes, we established the immune-related competing endogenous RNA network consisting of 33 lncRNAs, 21 miRNAs and 135 mRNAs. Prognostic analysis was performed on the genes in the immune-related competing endogenous RNA network to screen out 15 lncRNAs, 2 miRNAs and 31 mRNAs with prognostic values.

**Conclusion:**

In summary, we identified a novel immune-related mRNA-miRNA-lncRNA competing endogenous RNA network associated with the prognosis of AML, which may contribute to better understanding of the development and progression of AML and to serve as novel therapeutic targets.

## Introduction

Acute myeloid leukemia (AML) is the most common type of acute leukemia in adults, caused by the clonal expansion of undifferentiated myeloid progenitor cells (1). Although most patients with AML can achieve complete remission by induction chemotherapy, the recurrence rate remains high and thus is the main factor affecting the outcomes of AML patients. Relapse often develops from minimal residual disease in the protective bone marrow (BM) microenvironment (2, 3). A comprehensive understanding of the BM microenvironment is conducive to the diagnosis and personalized treatment of AML (3).

Cytogenetics and molecular aberrations are the main factors for risk stratification in patients with AML (4). In addition, the BM microenvironment also plays a very important role in the homing and survival of leukemic cells (3). The BM microenvironment contains various components such as immune cells, stromal cells, endothelial progenitor cells, extracellular matrix, growth factors, and cytokines (5). The interaction between leukemic cells and BM microenvironment affects resistance to chemotherapy in AML (3). The modulation of BM microenvironment in AML is currently undergoing preclinical research and early clinical trials. Molecular inhibitors such as CXCR4 inhibitors, VLA-4 inhibitors and E-selectin inhibitors are currently undergoing clinical trials (6, 7). Immune cells and stromal cells are important components of the BM microenvironment, and are also the main influencing factors of leukemia development (8, 9). The ESTIMATE program is a common method to explore the microenvironment of many tumors (10). Recently, it has also been used to explore the prognostic genes in the microenvironment of AML patients (11–13). Most studies have focused on differentially expressed genes (DEGs). However, the interaction and relationship between genes are open to investigate. Moreover, the coding genes were extensively explored, but regions that encoded lncRNAs and miRNAs were less well-studied.

Weighted gene coexpression network analysis (WGCNA) is an algorithm commonly used in systems biology to explore the correlation between gene sets and the clinic (14). Functionalization is achieved by constructing a free-scale coexpression gene network (14). WGCNA can identify highly related genes and aggregate them into the same genetic module. Currently, WGCNA is used in multiple fields such as cancer and nervous system, or to identify potential biomarker candidates or new therapeutic targets from genetic data (15–17).

The competition endogenous RNA (ceRNA) hypothesis was a new regulatory mechanism between non-coding RNA (ncRNA), and messenger RNA (mRNA) proposed by Salmena et al. in 2011 (18). In this theory, crosstalk between ceRNAs is achieved by competitively combining shared miRNAs (19). In recent years, the ceRNAs hypothesis has attracted widespread attention in the study of molecular and biological mechanisms of tumorigenesis and development (18). For example, previous studies have found that lncRNA-related ceRNAs were involved in the biological processes of glioblastoma and breast cancer (15, 20). The research on ceRNAs of leukemia was generally based on the differential genes screened by leukemia and normal controls (21), but no known module based on ceRNAs network related to microenvironment in leukemia has been set up.

In this study, mRNAs, miRNAs and lncRNAs data and clinical information of the AML cohort from The Cancer Genome Atlas (TCGA) database were used to calculate the immune and stromal scores of these AML cases using the ESTIMATE algorithm. Differentially expressed mRNAs and lncRNAs were applied to WGCNA to identify the modules most relevant to the AML immune microenvironment. Then the immune-related lncRNA-miRNA-mRNA ceRNA network was established to screen genes with clinical significance. These findings will help to better understand the role of tumor microenvironment in AML and shed light on the development and progression of AML.

## Materials and Methods

### Data Acquisition

All data sets of AML patients were downloaded from TCGA database^1^. The data used in this study met the following criteria: (1) Excluding samples combined with other malignancies; (2) Samples with lncRNAs and miRNAs and mRNAs detection data. Finally, all lncRNAs, mRNAs, and miRNAs expression profiles of 138 AML specimens and the corresponding clinical follow-up data were downloaded.

### Identification of Differentially Expressed Genes

The ESTIMATE algorithm^2^ was used to calculate the immune scores and stromal scores of 138 AML samples. Differentially expressed genes (DEGs), such as lncRNAs, miRNAs, and mRNAs, were identified between high and low score groups stratified by the median value of immune scores and stromal scores using limma package (22). All q values use FDR to correct the statistical significance of the multiple test. LncRNAs and mRNAs with |log FC| > 1.3 and FDR < 0.05 were regarded as differentially expressed while miRNAs with |log FC| > 2 and FDR < 0.05 were regarded as differentially expressed. Then all the DEGs were entered into R (version 3.5.1, Auckland, NZ, United States) for cluster analysis based on the expression value of each sample in its respective data set. The results were expressed in a clustergram. Each column represents a sample, and each row represents the expression level of a given gene.

### GO and Pathway Enrichment Analyses

The Database for Annotation, Visualization and Integrated Discovery (DAVID)^3^ was applied to analyze enriched biological themes of DEGs functions, particularly GO (gene ontology) terms and KEGG (Kyoto Encyclopedia of Genes and Genomes) pathway enrichment (23). *P* < 0.05 was set as the cut-off criterion.

### Weighted Gene Coexpression Network Analysis

WGCNA is an algorithm for identification of gene coexpression networks through high-throughput expression profiles of mRNAs or lncRNAs with different characteristics (14). Pairwise Pearson correlation analysis was used to evaluate the weighted coexpression relationship between all dataset topics in the adjacency matrix. In this study, WGCNA was used to analyze mRNAs and lncRNAs to obtain the mRNAs or lncRNAs most relevant to AML immune microenvironment.

### ceRNA Network Construction and Analysis

According to the results of WGCNA, we selected all mRNAs and lncRNAs in the most relevant module (turquoise) and differentially expressed miRNAs to construct a ceRNA network. Briefly, the associated ceRNA network in AML was constructed following three stages. (1) Prediction of lncRNA-miRNA: in order to make lncRNAs and miRNAs map into the interactions successfully, we used the miRanda^4^ and PITA^5^ to get targeted lncRNAs that miRNAs may regulate. (2) Prediction of miRNA-mRNA: three online databases, miRanda^4^, Targetscan^6^, and miRWalk^7^ were used simultaneously for target mRNA prediction. (3) Construction of lncRNA-miRNA-mRNA ceRNA network: the ceRNA network was constructed based on the negatively regulating target relationships of miRNA–mRNA and miRNA–lncRNA correlation pairs.

### PPI Network Construction

The Retrieval of Interacting Genes (STRING) database^8^ was utilized to construct a protein–protein interaction (PPI) network of the DEGs identified in the ceRNA network. The interacting pairs with a confidence score greater than 0.3 were considered as significant and were retained. The degree represents the number of interaction partners.

### Survival Analysis

Kaplan–Meier plots were constructed to illuminate the relationship between the overall survival of AML patients and the expression level of mRNAs, lncRNAs, and miRNAs. The statistical significance of the correlation was tested by the log-rank test and *P* < 0.05 was considered significant. The analysis was conducted using the R package of survival.

### Statistical Analysis

Graphpad Prism^TM^ 5.01 (San Diego, CA, United States) or R (version 3.5.1, Auckland, NZ, United States) software was used for all data analyses. Differences across groups were compared using *Kruskal–Wallis* test for continuous variables. Differences were considered significant when *P* < 0.05.

## Results

### Immune and Stromal Scores Are Associated With AML Clinical Parameters

We obtained gene expression profiles and clinical information of 138 AML patients from TCGA database (Supplementary Material 1). Among them, 74 (53.6%) were male and 64 (46.4%) were female, with a median age 55.5 (range 21–88) years old. According to the FAB classification, there were 13 cases of M0, 32 M1 cases, 34 M2 cases, 15 M3 cases, 27 M4 cases, 14 M5 cases, 2 M6 cases, and 1 M7 case. The ESTIMATE algorithm was used to calculate the immune scores and stromal scores of AML patients. The median immune score was 3027.01 (range from 1686.31 to 4291.79) and the median stromal score was −1024.97 (range from −1910.13 to 297.36).

**FIGURE 1:**
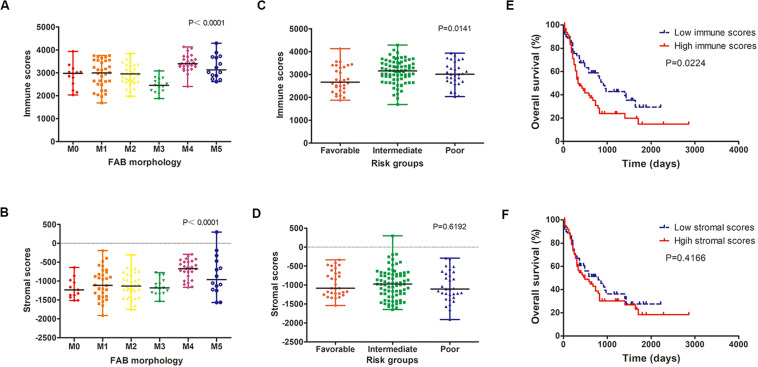
Immune and stromal scores are associated with AML clinical parameters. **(A,B)** Distribution of immune scores **(A)** and stromal scores **(B)** for AML FAB subtypes. **(C,D)** The correlation between immune scores **(C)** and stromal scores **(D)** and AML cytogenetic risk. **(E,F)** Kaplan–Meier survival curve based on immunes **(E)** and stromal scores **(F)**. AML, acute myeloid leukemia.

We analyzed the relationship between immune scores and stromal scores and clinical parameters of AML patients. Cases with M4 subtype AML had the highest immune scores while cases with M3 subtype had the lowest immune scores (*P* < 0.0001; Figure 1A). Similarly, M4 cases had the highest stromal scores, whereas M0 subtypes had the lowest (*P* < 0.0001; Figure 1B). According to cytogenetics, AML patients were divided into three groups: favorable, intermediate/normal and poor. There was an obvious correlation between the cytogenetic risk and the immune scores (*P* = 0.0141; Figure 1C; favorable *vs.* intermediate: *P* = 0.0041; favorable *vs.* poor: *P* = 0.1142; intermediate *vs.* poor: *P* = 0.2605), but no significant correlation between the cytogenetic risk and the stromal scores was observed (*P* = 0.6192; Figure 1D).

Using the median immune or stromal score as a threshold, AML patients were divided into two groups with low immune/stromal score and high immune/stromal score. Survival analysis showed that the survival rate of AML patients with low immune scores was significantly higher than that of patients with high immune scores (*P* = 0.0224; Figure 1E). However, there was no significant difference in survival between patients with low stromal scores and those with high stromal scores (*P* = 0.4166; Figure 1F).

### Identification of Differentially Expressed Genes Based on Immune Scores and Stromal Scores

**FIGURE 2:**
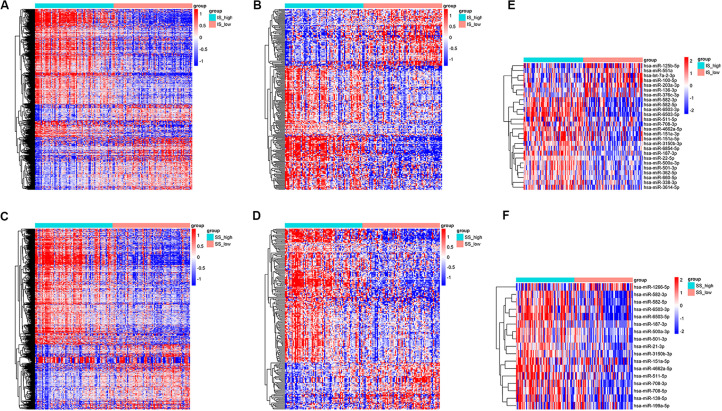
Heatmap of differentially expressed genes in the high and low immune/stromal score groups. **(A)** mRNAs, **(B)** lncRNAs and **(E)** miRNAs based on immune scores. **(C)** mRNAs, **(D)** lncRNAs, and **(F)** miRNAs based on stromal scores.

Setting the cut-off criteria as |log FC| > 1.3 and FDR < 0.05, we identified 1399 mRNAs (Figure 2A) and 258 lncRNAs (Figure 2B) based on immune scores, and 1166 mRNAs (Figure 2C) and 212 lncRNAs (Figure 2D) based on stromal scores. Setting the cut-off criteria as |log FC| > 2 and FDR < 0.05, we identified 26 and 17 miRNAs based on immune scores (Figure 2E) and stromal scores (Figure 2F), respectively. The DEGs of the low vs. high immune score or stromal score groups were illustrated in the heat map (Figure 2).

### Functional Enrichment Analysis of DEGs

**FIGURE 3:**
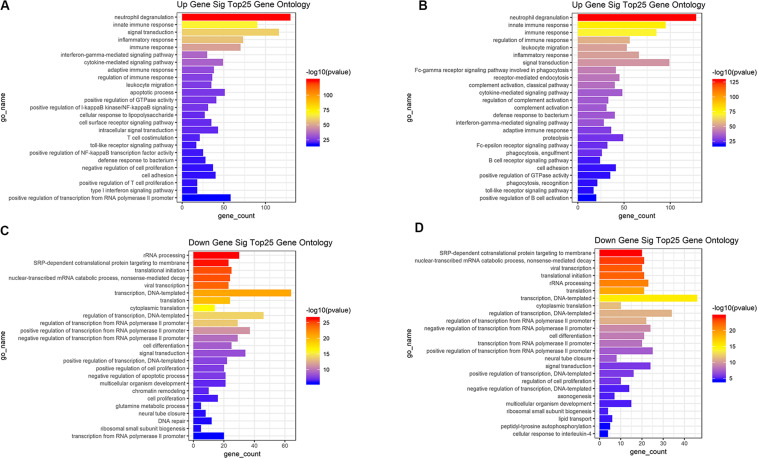
Top 25 GO terms in each of biological process were performed for functional enrichment clustering analysis. **(A)** Top25 significant GO terms based on upregulated genes in immune scores. **(B)** Top25 significant GO terms based on upregulated genes in stromal scores. **(C)** Top25 significant GO terms based on downregulated genes in immune scores. **(D)** Top25 significant GO terms based on downregulated genes in stromal scores. GO, gene ontology.

**FIGURE 4:**
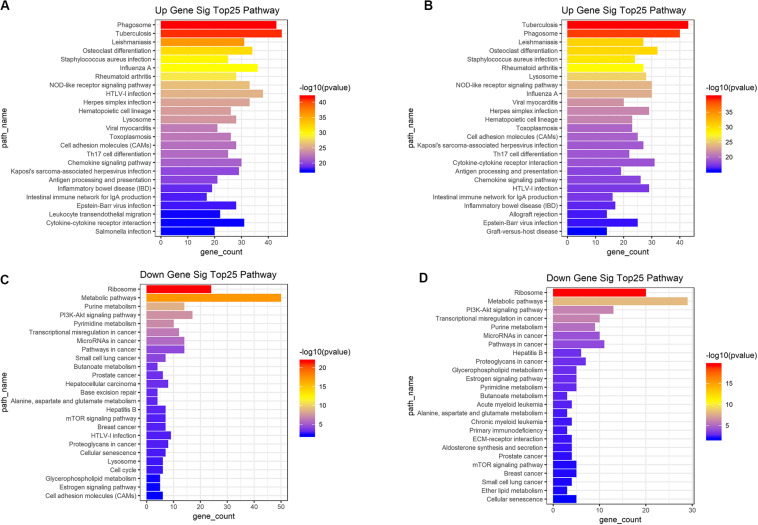
Top 25 KEGG pathway analysis were performed for functional enrichment clustering analysis. **(A)** Top25 significant KEGG pathways based on upregulated genes in immune scores. **(B)** Top25 significant KEGG pathways based on upregulated genes in stromal scores. **(C)** Top25 significant KEGG pathways based on downregulated genes in immune scores. **(D)** Top25 significant KEGG pathways based on downregulated genes in stromal scores. KEGG, Kyoto Encyclopedia of Genes and Genomes.

Based on the DAVID (The Database for Annotation, Visualization and Integrated Discovery) gene annotation tool, we performed GO analyses of both upregulated and downregulated DEGs. The top 25 GO biological process indicated that the upregulated DEGs based on immune or stromal scores were primarily enriched in neutrophil degranulation, regulation of immune response, signal transduction and inflammatory response (Figures 3A,B), while the downregulated DEGs based on immune/stromal scores were primarily enriched in rRNA processing, regulation of translation, regulation of transcription and cell differentiation (Figures 3C,D). Subsequently, we performed KEGG (Kyoto Encyclopedia of Genes and Genomes) pathway enrichment and interrelation analysis. KEGG analysis revealed that the upregulated DEGs were mainly enriched in infection, osteoclast differentiation, NOD-like receptor signaling pathway, hematopoietic cell lineage and cell adhesion molecules (CAMs) pathways (Figures 4A,B), while the downregulated DEGs were mainly enriched in ribosome, metabolism, PI3K-Akt signaling pathway, transcriptional dysregulation in cancer and miRNAs in cancer (Figures 4C,D). Above analyses revealed that these DEGs play a vital role in the development of AML and thus require further research to determine their biological contribution.

### Construction of Weighted Correlation Network Analysis and Identification of Key Modules

**FIGURE 5:**
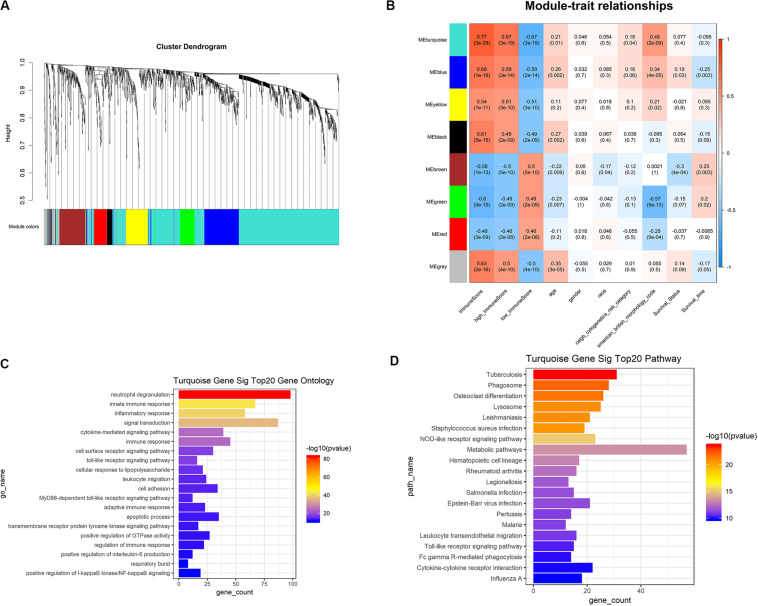
WGCNA was used to analyze genetic modules. **(A)** Cluster diagram of coexpression network modules based on topological overlap in mRNA and lncRNA. **(B)** Study the relationship between each module with their corresponding clinical characteristics. **(C)** GO analysis showed the gene symbols and gene interactions in the turquoise module. **(D)** KEGG analysis was used to study the pathway enrichment in the turquoise module. WGCNA, weighted correlation network analysis; GO, gene ontology; KEGG, Kyoto Encyclopedia of Genes and Genomes.

Based on the results of survival analysis, DEGs based on immune scores were selected for subsequent analysis. The best β value in the lncRNA/mRNA coexpression network was 5, which was calculated using the 258 differential lncRNAs, 1399 differential mRNAs, and their expression data in leukemia samples. Next, the method of dynamic tree cutting was used to produce coexpression modules. Finally, 8 modules of lncRNA-mRNA coexpression networks were generated and the heat map plot of topological overlap matrix (TOM) was shown (Figure 5A). Each module was calculated and plotted with its corresponding clinical characteristics. Correlation analysis showed that Turquoise module displayed the highest relationship with AML immune scores (*r* = 0.77), which included 760 mRNAs and 86 lncRNAs (Figure 5B). These 760 mRNAs were further used to perform the gene enrichment analysis. The genes were most related to neutrophil degranulation, immune response, inflammatory response, signal transduction and toll-like receptor signaling pathway (Figure 5C). In addition, genes were highly enriched in infection, osteoclast differentiation, NOD-like receptor signaling pathway, metabolic pathways and hematopoietic cell lineage by KEGG analysis (Figure 5D).

### ceRNA Network Construction

**FIGURE 6:**
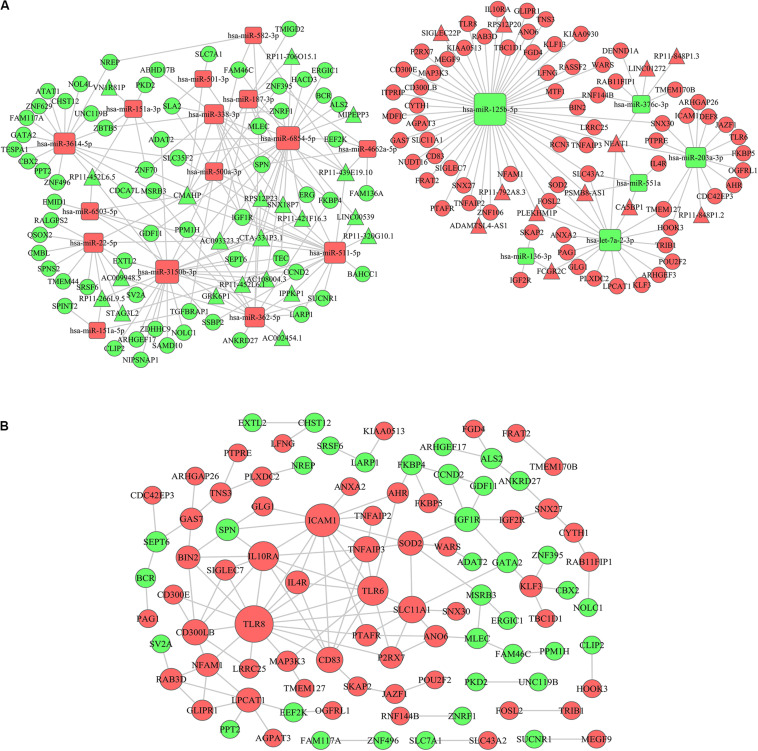
ceRNA network construction and protein–protein interaction network. **(A)** A lncRNA-miRNA-mRNA ceRNA network was constructed by 33 lncRNAs, 21 miRNAs, and 135 mRNAs. **(B)** A PPI network was constructed based on the STRING database. The rectangle represents microRNAs, the circle represents mRNAs and the triangle represents lncRNAs; the red represents up-regulation in IS-high and the green represents down-regulation in IS-high; the size of the dot represents the regulatory capacity of the mRNA, and larger points indicate stronger regulatory capability. ceRNA, competing endogenous RNAs; PPI: protein–protein interaction.

Since the Turquoise module showed the highest relationship with AML immune scores, we selected lncRNAs and mRNAs in the Turquoise module and 26 differentially expressed miRNAs based on immune scores to construct a ceRNA network. Firstly, based on the PITA and miRcode online database that matches potential miRNAs with lncRNAs, a total of 315 lncRNA-miRNA pairs contained 63 lncRNAs and 25 miRNAs. Then, we searched for the mRNAs targeted by the differentially expressed miRNAs using three target gene prediction websites, miRanda, miRWalk, and TargetScan. Using these websites, we detected 664, 671, and 655 target mRNAs, respectively. Based on the Venn intersection analysis, 289 target mRNAs were selected. Subsequently, we matched the predicted target genes with the mRNAs in the Turquoise module. Then, we constructed the ceRNA network by integrating the miRNA-lncRNA-mRNA interactions. At last, a final lncRNA-miRNA-mRNA ceRNA network was constructed with 33 lncRNAs, 21 miRNAs and 135 mRNAs (Figure 6A).

### Protein–Protein Interaction (PPI) Network Analysis

To further explore the interplay among the mRNAs in ceRNA, we constructed a PPI network based on the STRING (The Retrieval of Interacting Genes) online database (Figure 6B). In the network, *TLR8* (Toll Like Receptor 8), *ICAM1* (Intercellular Adhesion Molecule 1), *TLR6* (Toll Like Receptor 8), and *IL10RA* (Interleukin 10 Receptor Subunit Alpha) had higher degrees (16, 13, 10, and 10, respectively) (Supplementary Table 1). The genes encoding these proteins have been confirmed to be associated with immune microenvironment and leukemia progression (24–27).

### Association Between mRNAs, miRNAs, and lncRNAs in ceRNA and Overall AML Survival

**FIGURE 7:**
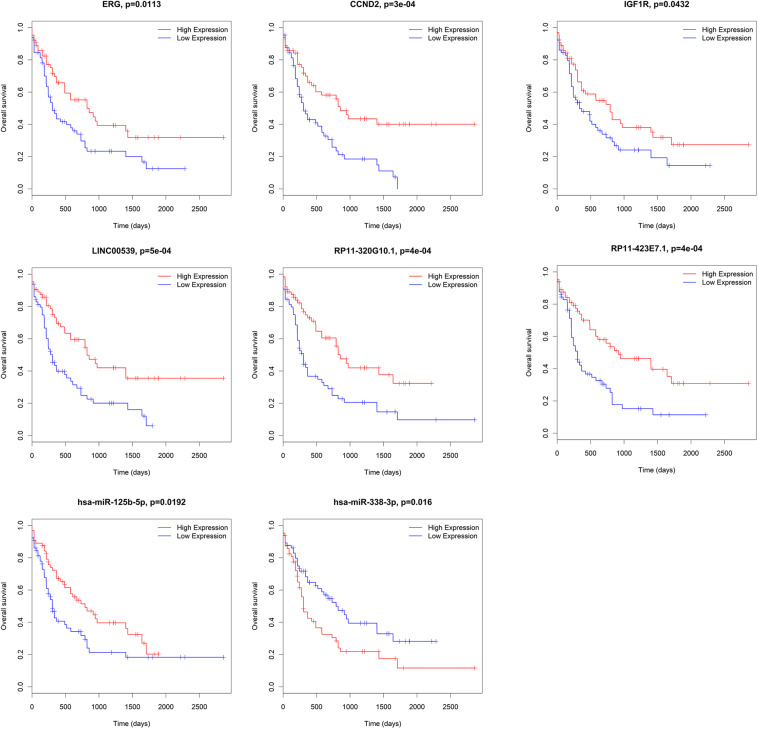
Correlation between mRNAs, miRNAs and lncRNAs in ceRNA and overall AML survival in TCGA. Kaplan–Meier survival curves with the log-rank test were performed for the representative mRNAs, miRNAs and lncRNAs. ceRNA, competing endogenous RNAs; AML, acute myeloid leukemia; TCGA: The Cancer Genome Atlas.

We further analyzed the prognostic values of mRNAs, miRNAs, and lncRNAs in the ceRNA network. Subjects were divided into high-expression and low-expression cohorts according to the median value of these genes. For overall survival, the high-expression and low-expression cohorts were split for the log-rank test through the package survival in R software. 15 out of 33 lncRNAs (*AC009948.5, CMAHP, CTA-331P3.1, FCGR2C, GRK6P1, LINC00539, LINC01272, MIPEPP3, PSMB8-AS1, RP11-266L9.5, RP11-320G10.1, RP11-421F16.3, RP11-439E19.10, RP11-792A8.3* and *STAG3L2*), 2 out of 21 miRNAs (*hsa-miR-125b-5p* and *hsa-miR-338-3p*) and 31 out of 135 mRNAs (*AGPAT3, ANKRD27, CBX2, CCND2, CD300LB, CYTH1, ERG, GDF11, IGF1R, KIAA0513, KIAA0930, LARP1, LFNG, LPCAT1, NREP, NUDT16, POU2F2, PPM1H, PTAFR, QSOX2, RAB3D, RALGPS2, SIGLEC7, SLC43A2, SRSF6, TNFAIP2, TNS3, TRIB1, ZBTB5, ZNF70*, and *ZNRF1*) were associated with overall survival according to the log-rank test (*P* < 0.05). Representative genes are shown in Figure 7.

Age (≥60 years vs. <60 years) and risk group (favorable *vs.* intermediate/normal *vs.* poor) were also associated with overall survival according to the log-rank test (*P* = 0.000 and 0.002, respectively). The above mentioned 15 lncRNAs, 31 mRNAs and 2 miRNAs were brought into further multivariate cox proportional hazard regression analysis with age and risk group. Finally, 7 mRNAs (*CCND2, ERG, LPCAT1, NUDT16, RALGPS2, TNFAIP2* and *ZNF70*), 6 lncRNAs (*CMAHP, FCGR2C, PSMB8-AS1, RP11-266L9.5, RP11-320G10.1, RP11-792A8.3*, and *STAG3L2*) and 2 miRNAs (*hsa-miR-125b-5p* and *hsa-miR-338-3p*) were independently associated with overall survival (Table 1).

**TABLE 1:** Multivariate cox proportional hazard regression analysis of 6 lncRNAs, 7 mRNAs, and 2 miRNAs.

Genes	HR (95%CI)	*P*
*CCND2*	0.640 (0.373–0.977)	0.040
*ERG*	0.603 (0.377–0.967)	0.036
*LPCAT1*	1.888 (1.179–3.023)	0.008
*NUDT16*	1.826 (1.143–2.917)	0.012
*RALGPS2*	0.414 (0.258–0.663)	0.000
*TNFAIP2*	2.010 (1.263–3.199)	0.003
*ZNF70*	0.526 (0.328–0.842)	0.007
*CMAHP*	0.580 (0.367–0.918)	0.020
*FCGR2C*	1.741 (1.103–2.748)	0.017
*PSMB8-AS1*	1.776 (1.116–2.828)	0.015
*RP11-266L9.5*	0.597 (0.373–0.957)	0.031
*RP11-320G10.1*	0.540 (0.338–0.862)	0.010
*RP11-792A8.3*	0.608 (0.390–0.948)	0.028
*STAG3L2*	0.597 (0.375–0.950)	0.030
*hsa-miR-125b-5p*	0.562 (0.353–0.897)	0.016
*hsa-miR-338-3p*	1.609 (1.010–2.564)	0.045

## Discussion

In recent years, studies about the roles of gene mutations and chromosomal translocations in the occurrence and development of AML and their prognostic values have made significant progress (4). However, the BM microenvironment which also plays an important role in the pathophysiology process in AML are poorly understood (5). Therefore, most treatments previously targeted only tumor cells, but few targeted the tumor microenvironment. In-depth research on the microenvironment of leukemia will help to further understand the mechanism of leukemia development, and may find new targets for microenvironment treatment (7). This study screened microenvironment-related genes based on the TCGA database, and further established microenvironment-related lncRNA-miRNA-mRNA ceRNA networks through WGCNA.

First, we calculated the immune scores and stromal scores of AML patients based on the ESTIMATE algorithm, and found that these scores were related to the FAB typing of AML. In addition, immune scores were significantly correlated with cytogenetic risk and overall survival. ESTIMATE is a new algorithm to infer the level of stromal and immune cells in tumor tissues and tumor purity using gene expression data (10). High immune scores in the BM samples from patients with poor prognosis indicated that more immune cells were recruited in their BM microenvironment. This may be due to that AML cells actively shape the BM environment and immune cells to promote disease progression through cellular, structural, and functional changes (11). However, there was no significant correlation between stromal scores and cytogenetic risk or survival of AML patients, suggesting that the proportion of stromal cells were comparable in different group. Possible explanation may be that stromal cells play an important role in solid tumors (28, 29), while its role in leukemia is not as strong as in solid tumors (11–13).

Then we identified differentially expressed mRNAs, miRNAs, and lncRNAs based on the immune scores or stromal scores. Functional enrichment analysis indicated that these DEGs were mainly involved in immune and inflammatory responses. Consistent with these results, previous studies have shown that the biology of the immune system is essential for the formation of a complex BM microenvironment (5). In recent years, the understanding of the immunological characteristics of AML has increased, and the development of effective AML immunotherapy strategies has attracted widespread attention (30).

In recent years, important advances in ceRNA coexpression network research have developed rapidly. The disruption of the ceRNA network balance is a major cause for tumorigenesis (31). Therefore, understanding the complex interactions between different ceRNA networks will lead to an in-depth understanding of gene regulatory networks and has implications for cancer treatment (31). In addition, the lncRNA-miRNA-mRNA ceRNA network can predict the prognosis of the disease. For example, a lncRNA-miRNA-mRNA ceRNA network was established based on RNA-Seq data of breast cancer from TCGA, which consists of 8 miRNAs, 48 lncRNAs, and 10 mRNAs (32). Multiplex cox regression analyses showed that four of these lncRNAs (*ADAMTS9-AS1, LINC00536, AL391421.1*, and *LINC00491*) have significant prognostic value (32). Wang et al. identified 108 lncRNAs, 10 miRNAs, and 8 mRNAs from a database to construct a lncRNA-miRNA-mRNA ceRNA network (21). In the network, a univariate and multivariate cox proportional hazard regression analysis was used to establish a survival model with 8 target mRNAs (*HOXA9, INSR, KRIT1, MYB, SPRY2, UBE2V1, WEE1*, and *ZNF711*), where AUC (area under curve) is 0.831, indicating the sensitivity and specificity of prognostic prediction (21). However, the screening of differential genes in this study was based on leukemia patients and normal people, and did not focus on tumor microenvironment. So far, there is no ceRNA research based on the leukemia microenvironment. In our research, the screening of differential genes was based on the immune score. Then WGCNA was used to identify the modules most relevant to the AML immune microenvironment. Then, using WGCNA and miRNA prediction websites, a lncRNA-miRNA-mRNA ceRNA network consisting of 33 lncRNAs, 21 miRNAs and 135 mRNAs was constructed.

Subsequently, we built a PPI network predicting the interaction among the proteins encoded by the 135 DEGs in the ceRNA network. *TLR8*, *ICAM1*, *TLR6*, and *IL10RA* had higher degrees. *TLR8* and *TLR6* are members of the Toll-like receptor family which is upstream to the transcription factor NFκB and part of the innate immune system and plays an important role in progression of AML (24). *ICAM1* is one of the CAMs, a large class of transmembrane proteins, involved in the binding of cells to another cell or extracellular matrix, and involved in cell proliferation, differentiation, movement, transportation, apoptosis and tissue structure (25). The protein encoded by *IL10RA* is a receptor for interleukin 10, which has been shown to mediate the immunosuppressive signal of interleukin 10, and thus inhibits the synthesis of proinflammatory cytokines and is reported to promote survival of progenitor myeloid cells through the insulin receptor substrate-2/PI3K/AKT pathway (26, 27). These results indicated that this novel ceRNA network were closely associated with immune microenvironment and progression of AML.

Furthermore, 15 lncRNAs, 2 miRNAs, and 31 mRNAs with prognostic significance were screened out, which could be used as biomarkers for prognosis. Among the genes with prognostic significance in our module of immune-related ceRNA network, there were AML related reports about *CBX2*, *CCND2, ERG, IGF1R, LARP1, LFNG, NUDT16, POU2F2, PTAFR, RAB3D, SIGLEC7, SRSF6, TNFAIP2, TRIB1, ZBTB5*, and *ZNRF1*, the most reported of which were *ERG, CCND2*, and *IFG1R*. ERG translocation was involved in the occurrence and development of AML, and high expression of ERG was a poor prognostic factor for patients with normal karyotype AML (33, 34). CCND2 mutations were more common in CBF-AML, and it was also a frequent mutation event in *t* (8, 21) AML (35, 36). CCND2 leaded to increased phosphorylation of retinoblastoma proteins, leading to significant cell cycle changes and increased proliferation of AML cell lines (36). Nicolas Chapuis et al. found that IGF-1 spontaneous lesions played a key role in PI3K/AKT activation of AML cells, providing strong evidence for targeting IGF1R as a potential new therapy for AML (37). The functions of *AGPAT3, ANKRD27, CD300LB, CYTH1, GDF11, KIAA0513, KIAA0930, LPCAT1, NREP, PPM1H, QSOX2, RALGPS2, SLC43A2, TNS3*, and *ZNF70* in AML have not been reported. We identified 15 lncRNAs with clinical significance. Among them, only *CMAHP* was reported to be related to MLL-positive AML (38), and other lncRNAs have not been reported in leukemia. Two miRNAs, including *hsa-miR-125b-5p* and *hsa-miR-338-3p*, have been reported to be associated with a variety of cancers (39–42), but no studies have been reported related to AML. All these unreported mRNAs, miRNAs and lncRNAs may be potential novel biomarkers or therapeutic targets for AML.

It is important to note that our current research has limitations. We selected the target data from the TCGA public database only through the biological algorithm method. We should further verify the results of this article in clinical patients in further study.

## Conclusion

In summary, a comprehensive bioinformatics analysis was performed on the AML dataset in TCGA, with an emphasis on the immune microenvironment. Using WGCNA and miRNA prediction programs, an immune-related lncRNA-miRNA-mRNA ceRNA network was established, and DEGs with prognostic value were further identified. Further studies of these genes are needed in the clinic and may provide new insights into the pathogenesis of AML. This study increases our understanding of the complex interactions between AML tumor cells and the BM microenvironment and may provide novel prognostic factors and therapeutic targets.

## Data Availability Statement

All data sets of AML patients were downloaded from The Cancer Genome Atlas (TCGA) database (https://portal.gdc.cancer.gov/).

## Author Contributions

YFL, CW, and ZJ: conceptualization and design. SW: data acquisition and writing – original draft. SW, LY, YX, and DZ: methodology. SW and CW: data analysis and interpretation. YJL and YFL: writing – review and editing. YFL, CW, and ZJ: project administration. All authors contributed to the article and approved the submitted version.

## Conflict of Interest

The authors declare that the research was conducted in the absence of any commercial or financial relationships that could be construed as a potential conflict of interest.

## Footnotes

1 https://portal.gdc.cancer.gov/

2 https://sourceforge.net/projects/estimateproject/

3 http://david.ncifcrf.gov/

4 http://www.microrna.org/

5 https://genie.weizmann.ac.il/pubs/mir07/mir07_exe.html

6 http://www.targetscan.org/

7 http://129.206.7.150/

8 http://string-db.org

## Abbreviations

AML: acute myeloid leukemia
AUC: area under curve
BM: bone marrow
CAM: cell adhesion molecules
ceRNA: competing endogenous RNAs
DEG: differentially expressed gene
DAVID: The Database for Annotation Visualization and Integrated Discovery
GO: gene ontology
ICAM1: Intercellular Adhesion Molecule 1
IL10RA: Interleukin 10 Receptor Subunit Alpha
KEGG: Kyoto Encyclopedia of Genes and Genomes
PPI: protein-protein interaction
TCGA: The Cancer Genome Atlas
TLR6: Toll Like Receptor 8
TLR8: Toll Like Receptor 8
TOM: topological overlap matrix
WGCNA: weighted correlation network analysis.
