# Supplementary material for: A Novel Immune-Related Competing Endogenous RNA Network Predicts Prognosis of Acute Myeloid Leukemia
Source: Front Oncol. 2020 Aug 11;10:1579. doi: 10.3389/fonc.2020.01579 (PMC7432272; doi:10.3389/fonc.2020.01579)
Supplement: Supplementary file 1 [file Table_1.docx]

Supplementary Table 1. Top 20 degree of genes in PPI network.

| Gene symbol | Biotype | Degree |
| --- | --- | --- |
| TLR8 | mRNA | 16 |
| ICAM1 | mRNA | 13 |
| TLR6 | mRNA | 10 |
| IL10RA | mRNA | 10 |
| SLC11A1 | mRNA | 8 |
| CD83 | mRNA | 7 |
| CD300LB | mRNA | 7 |
| TNFAIP3 | mRNA | 7 |
| IGF1R | mRNA | 6 |
| LPCAT1 | mRNA | 6 |
| SOD2 | mRNA | 6 |
| BIN2 | mRNA | 5 |
| IL4R | mRNA | 5 |
| NFAM1 | mRNA | 5 |
| P2RX7 | mRNA | 4 |
| PTAFR | mRNA | 4 |
| KLF3 | mRNA | 4 |
| RAB3D | mRNA | 4 |
| ANO6 | mRNA | 4 |
| GAS7 | mRNA | 4 |
